# Supplementary material for: Identification and functional analysis of non-coding regulatory small RNA FenSr3 in Bacillus amyloliquefaciens LPB-18
Source: PeerJ. 2023 May 15;11:e15236. doi: 10.7717/peerj.15236 (PMC10194069; doi:10.7717/peerj.15236)
Supplement: Supplemental Information 4 [file peerj-11-15236-s004.zip › KO/CK-vs-T1_map/map04966.html]

KEGG PATHWAY: Collecting duct acid secretion - Reference pathway


|  |  |
| --- | --- |
| **Collecting duct acid secretion - Reference pathway** |  |

[
Pathway menu
| Organism menu
| Pathway entry
| Show description
| User data mapping
]

One of the important roles of the collecting duct segment of the kidney nephron is acid secretion. As daily food intake loads acid into the body, urinary acid excretion is essential, and urine pH can drop as low as 4.5. The alpha-intercalated cell of collecting duct is the main responsible for hydrogen secretion into the urine. The carbon dioxide, which is generated in the cells and enters from the blood, is changed to carbonic acid. This carbonic acid is divided into hydrogen ion and bicarbonate ion. Intracellular CA II catalyses the formation of these ions. The hydrogen ion is secreted into the lumen by the luminal H(+)-ATPase. The bicarbonate ion is transported to the blood side by the anion exchanger type 1. Hydrogen ion in the lumen is trapped by urinary buffers. These include ammonium and phosphate.

|  |  |  |
| --- | --- | --- |
| Reference pathway | 184% 150% 122% 100% 82% 67% 55% | 图片下载 |
